# The role and mechanism of HMGB1-mediated Notch1/Hes-1 pathway in anxiety and depression-like behaviors in mice with chronic rhinosinusitis

**DOI:** 10.1186/s10020-024-01057-6

**Published:** 2025-01-09

**Authors:** Fangwei Zhou, Yiting Jiang, Yangsong Li, Jianyao Li, Tian Zhang, Guodong Yu

**Affiliations:** 1 Department of Otorhinolaryngology Head and Neck Surgery, Affiliated Hospital of Guizhou Medical University, Guiyang, 550004 China; 2 Guizhou Research Institute for Health Development, Guizhou Medical University, Guiyang, 550004 China

**Keywords:** Chronic rhinosinusitis, Anxiety and depression-like behaviors, HMGB1, Microglia, Metformin

## Abstract

**Background:**

Chronic rhinosinusitis (CRS) is a global health issue, with some patients experiencing anxiety and depression-like symptoms. This study investigates the role of HMGB1 in anxiety and depression-like behaviors associated with the microglial Notch1/Hes-1 pathway in CRS mice.

**Methods:**

A CRS mouse model was developed, and behavioral assessments were conducted to evaluate anxiety and depression-like behaviors. Techniques including ^18^F-FDG PET, Nissl staining, and immunofluorescence were used to assess hippocampal metabolic activity in CRS mice. Western Blot and RT-qPCR were employed to measure HMGB1 and Notch1/Hes-1 expression in the hippocampus, while ELISA determined inflammatory cytokine levels. The study also examined the effects of metformin on these behaviors and its mechanisms.

**Results:**

CRS mice exhibited increased anxiety and depression-like behaviors, accompanied by enhanced hippocampal metabolic activity. HMGB1-siRNA treatment reduced these behaviors. Hippocampal glucose metabolism was markedly higher in CRS mice than in controls. Nissl staining revealed hippocampal neuron damage, and immunofluorescence indicated microglial activation in CRS mice. Reducing HMGB1 expression inhibited Notch1/Hes-1 pathway activation. In microglia, HMGB1 knockdown suppressed the Notch1/Hes-1 pathway, reducing inflammatory cytokine secretion. Metformin improved neuropsychiatric symptoms in CRS mice by inhibiting the Notch1/Hes-1 pathway after HMGB1 downregulation.

**Conclusion:**

HMGB1 activates the microglial Notch1/Hes-1 pathway in CRS mice, promoting neuroinflammation and anxiety and depression-like behaviors. Metformin alleviates these effects.

## Introduction

Chronic rhinosinusitis (CRS) is one of the most common chronic inflammatory diseases of the upper respiratory tract, characterized by persistent symptoms like nasal obstruction, rhinorrhea, headache, and hyposmia for over 12 weeks (Cho et al. 2020). CRS significantly impacts patients’ daily lives and work performance, and it places a considerable strain on medical resources, thereby imposing a heavy burden on both individuals and society (Cho et al. 2020; Bachert et al. 2021). CRS is also recognized for its association with mental health issues. Anxiety and depression are common psychological states that can influence patients’ perception of the burden of chronic illnesses such as CRS and may further impact the degree of improvement following surgical intervention (Ghadami 2019). In the general population, the prevalence of depression ranges from 2 to 14%, but it is higher among CRS patients, approximately 20–25% (Tomoum et al. 2015). According to relevant reports, CRS patients with olfactory dysfunction or asthma are more prone to anxiety and depressive symptoms, and olfactory dysfunction is a notable risk factor that significantly affects the mental health and quality of life (Soy et al. 2013). Although previous studies have reported on the phenomenon of CRS accompanied by anxiety and depression, the underlying mechanisms remain unclear.

Microglia, the primary immune cells of the central nervous system (CNS), extensively present across the brain, spinal cord, and brainstem, playing a crucial role in immune surveillance and forming the CNS’s first line of defense (Prinz et al. 2019). They activate in response to inflammation, ischemia, infection, and neurodegenerative diseases (Xu et al. 2016). Research indicates that microglia are implicated in the pathophysiology of anxiety and depression, primarily by modulating CNS inflammatory responses, the hypothalamic-pituitary-adrenal axis, and gut microbiota, which contribute to the development of these conditions (Nayak et al. 2014). In animal models of anxiety and depression, activated microglia are abundant in the hippocampus, prefrontal cortex, amygdala, and paraventricular nucleus, correlating positively with the severity of the conditions (Wohleb 2016). The hippocampus, crucial for memory and spatial orientation, plays a central role in emotional memory formation, regulation, and cognition (Ekstrom and Ranganath 2018; Anacker and Hen 2017). Its dysfunction may link to emotional disorders such as anxiety and depression. Hippocampal functional abnormalities could lead to emotional dysregulation, potentially causing anxiety and depression, with the abnormal activation of microglia in this region possibly being a key mechanism in CRS patients who also suffer from anxiety and depression.

High mobility group protein 1 (HMGB1), a nuclear DNA-binding protein broadly presented in eukaryotic cells, is secreted by cells in response to stress stimuli, either actively or passively from necrotic cells, to exert pro-inflammatory effects (Li et al. 2023; Chen et al. 2018). Elevated HMGB1 levels in the nasal secretions of CRS patients are linked to disease exacerbation and lesion expansion (Min et al. 2015). Taziki et al. (Taziki et al. 2019) found significantly higher HMGB1 expression in the serum and nasal mucosa of CRS patients compared to the general population. HMGB1 directly stimulates peripheral inflammatory cells to release factors, contributing to nasal inflammation and playing a key role in CRS pathogenesis (Ciprandi et al. 2020). Additionally, HMGB1 is implicated in cognitive dysfunction and neurodegenerative diseases (Fang et al. 2012). Chibaatar et al. (Chibaatar et al. 2021) demonstrated that melatonin can inhibit microglia-induced neuroinflammation by downregulating HMGB1 expression. HMGB1’s association with microglial activation is crucial in neuroabnormalities and cognitive dysfunction related to central inflammation (Yin et al. 2023). Abnormal HMGB1 expression is also closely related to anxiety and depression (Lu et al. 2024; Xu et al. 2020), suggesting that HMGB1-induced microglial activation may be a significant factor in the development of anxiety and depression in CRS patients.

Research indicates that the Notch signaling pathway regulates cell development and differentiation and also drives inflammation (Webb and Tait Wojno 2019). Hes-1, a Notch1 downstream effector, promotes the release of inflammatory factors and is associated with various inflammatory responses (Shang et al. 2016). Inhibiting Notch1 expression in microglia can reduce microglial activation and alleviate neuroinflammation (Chen et al. 2023). In mice with acute infarction, injecting exogenous HMGB1 activates genes and signaling pathways crucial for cardiomyocyte survival and regeneration, a process linked to Notch pathway activation in cardiomyocytes (Limana et al. 2013). In a rat model of ischemia-reperfusion, activated microglia produce more inducible nitric oxide synthase and reactive oxygen species, with increased Notch1 and Hes-1 expression (Zeng et al. 2017). Treating BV-2 microglia with LPS activates the Notch pathway, significantly increasing the expression of Notch1, Hes-1, tumor necrosis factor (TNF-α), and IL-12 (Wu et al. 2018). It remains unclear if HMGB1 can promote inflammatory factor release and neuroinflammation by regulating the Notch1/Hes-1 pathway.

Metformin, classified as a biguanide medication, is predominantly employed in the management of type 2 diabetes (Foretz et al. 2014). It has multiple biological effects, such as inhibiting inflammation, preventing apoptosis, reducing oxidative stress, and exhibiting anti-tumor activity (Ren et al. 2020). Metformin is capable of penetrating the blood-brain barrier, providing anti-inflammatory and neuroprotective benefits in various CNS disease models (Agostini et al. 2021; Dutta et al. 2023). Studies suggest metformin’s potential in anti-anxiety and antidepressant therapies, though its mechanisms are not fully understood (Zemdegs et al. 2019). Its demonstrated anti-anxiety and antidepressant effects make it a promising option for diabetics with comorbid anxiety and depression (Fung et al. 2018). Metformin also alleviates depressive symptoms in rats under chronic unpredictable stress (Fang et al. 2020). Further research into metformin’s therapeutic targets and pathways could guide treatments for anxiety and depression, especially in CRS patients.

Drawing on prior research, our investigation will focus on the effects of HMGB1 and the Notch1/Hes-1 pathway on anxiety and depression symptoms in CRS, as well as their mechanisms within hippocampal microglia. Additionally, the potential therapeutic role of metformin will be evaluated. This research aims to elucidate the underlying molecular mechanisms and signaling pathways, establishing a theoretical foundation for considering HMGB1 as a novel therapeutic target for CRS-related anxiety and depression and providing clear guidance for future clinical applications.

## Materials and methods

### Animals

C57BL/6 female mice, aged 6 to 8 weeks, were sourced from the Experimental Animal Center of Guizhou Medical University. Mice were housed in a specific-pathogen-free environment at standard temperature (18–22 °C), moderate humidity (50–55%), and under a 12 h light/dark cycle. All mice were acclimated in the animal room for 1 week before being used in experiments. All protocols for animal experiments were approved by the Experimental Animal Ethics Committee of Guizhou Medical University (No. 2201201).

### Murine CRS model and related experiments

The research was divided into three separate experiments.

**Fig. 1:**
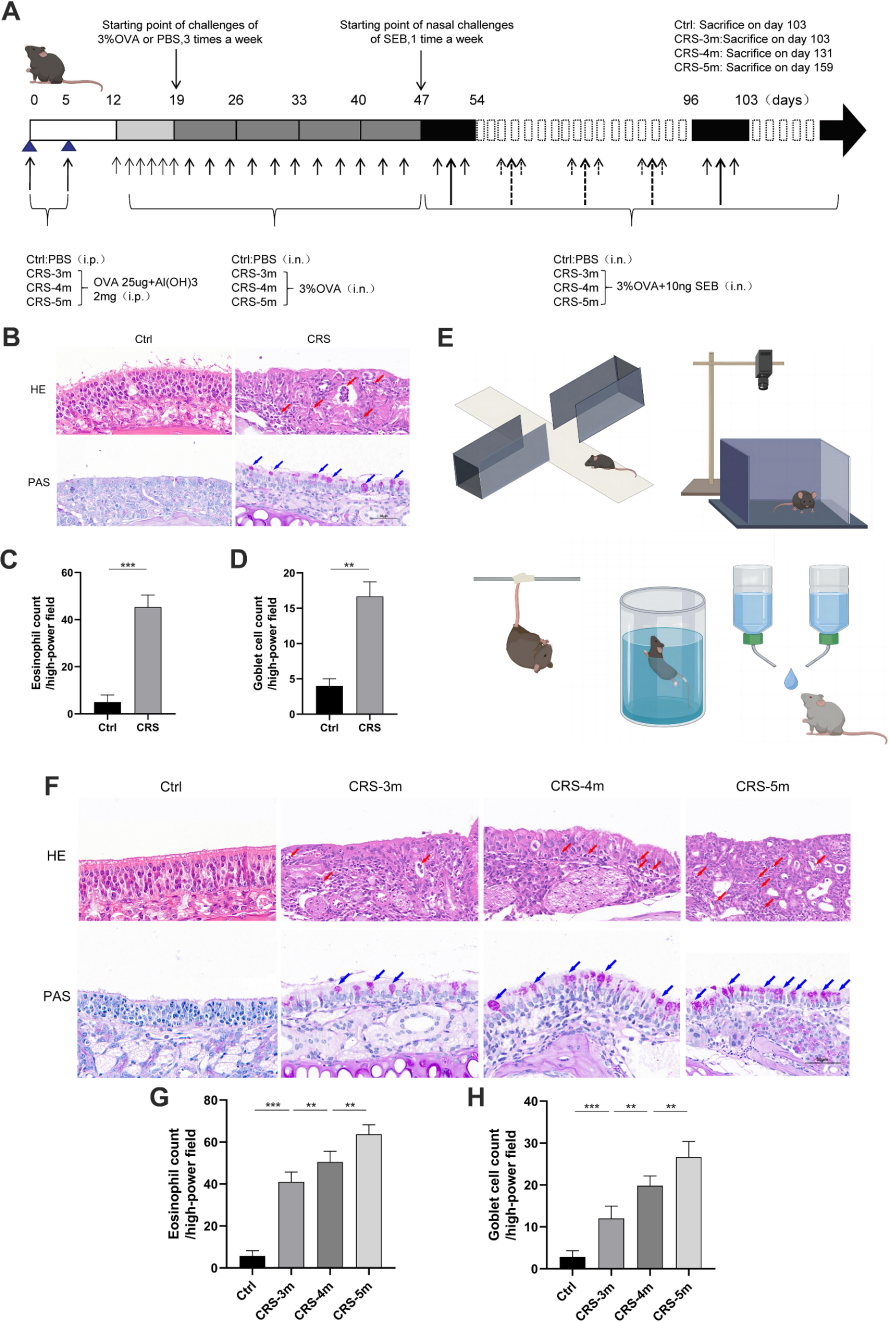
Varying inflammation in established CRS mouse models. (**A**) Experimental procedure timeline. (**B**) HE and PAS staining of nasal mucosa from Ctrl and CRS groups (×400). (**C**) Eosinophil count in nasal mucosa. (**D**) Goblet cell count in nasal mucosa. (**E**) Behavioral experiment schematic. (**F**-**H**) Staining, eosinophil, and goblet cell counts for additional mouse groups (×400). Red arrows indicate eosinophils; blue arrows indicate goblet cells. Data are presented as mean ± SD. **P < 0.01, ***P < 0.001

In the first experiment, mice were assigned to four groups, each consisting of 10 mice: Control (Ctrl), CRS-3 m, CRS-4 m, and CRS-5 m. The experimental schedule is shown in Fig. 1A. A mouse model of CRS was created following an established method (Kim et al. 2011). In short, experimental group mice were sensitized by intraperitoneal (i.p.) injections of ovalbumin (25 mg, OVA, Sigma-Aldrich) in 200 µl of PBS with aluminum hydroxide (2 mg) on days 0 and 5. Subsequently, they underwent intranasal (i.n.) challenges with 20 µl of a 3% OVA solution for 13 weeks continuously. During the final 8 weeks, mice in the CRS-4 m and CRS-5 m groups (12 and 16 weeks respectively) were challenged with staphylococcal enterotoxin B (10 ng SEB, Toxin Technology, USA) and OVA via intranasal administration. The Ctrl group received PBS throughout the experiment for comparative purposes.

For the second experiment, the mice were assigned to the subsequent groups (*n* = 10 per group): Ctrl, CRS-3 m, CRS-3 m + siRNA-HMGB1, CRS-3 m + siRNA-NC. The experiment schedule is shown in Fig. 4A. The CRS-3 m + siRNA-HMGB1 and CRS-3 m + siRNA-NC groups followed a treatment process similar to the CRS-3 m group, with a key difference occurring on the 103rd day of the study. On this day, mice in both groups underwent stereotactic brain injections: the CRS-3 m + siRNA-HMGB1 group received siRNA-HMGB1, while the CRS-3 m + siRNA-NC group received siRNA-NC (negative control siRNA).

In the third experiment, the mice were allocated to the subsequent groups (*n* = 10 per group): Ctrl, CRS, CRS + Met, CRS + Saline. The experiment schedule is shown in Fig. 7A. After establishing the CRS model, the CRS + Met group received daily intraperitoneal injections of a metformin solution (200 mg/kg) for one week, while the CRS + Saline group received equivalent injections of saline solution.

After the completion of modeling and related interventions, mice underwent behavioral testing and PET experiments, followed by euthanasia and tissue collection.

### Stereotactic injection

After the establishment of the CRS mouse model, we used a mouse brain atlas to determine the location of the hippocampal region based on coordinates: 2.3 to 2.4 millimeters posterior from the suture, 1.5 to 1.6 millimeters lateral, and 2.0 millimeters in depth. Isoflurane was used to anesthetize the mice, after which the scalp was incised and a hole was drilled. Subsequently, we used a microsyringe to inject 2 microliters (concentration of 2.5 mM/L) of siRNA-HMGB1 or siRNA-NC (Genechem, China) into each side to achieve bilateral injections. After the injection, the incision is sutured. These are the primer sequences for siRNA-HMGB1, 5’ - UCUUGACCACAGAUCUUAATT − 3’ (forward) and 5’ - AGCCTTGTGTGTTTCTGCG − 3’ (reverse); for siRNA-NC, 5’ - CCAAGAACTTCCAGAACATAT − 3’ (forward) and 5’ - ATATGTTCTGGAAGTTCTTGG − 3’ (reverse).

### Behavioral experiment

#### Elevated plus maze

Comprising two enclosed arms and two open arms, each with dimensions of 30 cm long, 6 cm wide, and 15 cm tall, the apparatus is utilized to gauge the subjects’ anxiety levels. Animals are introduced to the maze and permitted to explore for a duration of 5 min, during which their duration of stay in both the enclosed and open arms is meticulously recorded.

#### Open field test

A simple apparatus, measuring 44 cm × 44 cm × 30 cm, was employed to evaluate the movement and anxiety-related behavior of the subjects. Following a brief 30-second acclimatization period, the total distance covered and the duration spent in the central area (14.7 cm × 14.7 cm) were measured over a 5-minute observation period.

#### Tail suspension test

Mice were subjected to a tail suspension test, where they were secured by tape 1 cm from the tail’s tip, suspended 15 cm above the ground. To prevent climbing, small plastic tubes were secured around their tails. The duration of the test was set at 6 min, with immobility being quantified and analyzed during the last 4 min of the session.

#### Forced swim test

Individual mice were placed into separate clear glass tubes, each 6 cm in diameter and 30 cm tall, containing water to a height of 18 cm, with the temperature maintained at approximately 25 °C. The rodents were observed within these tubes for a duration of 6 min, with the period of immobility being quantified in the final 4 min of the observation.

#### Sucrose preference test

Prior to the experiment, the mice were accustomed to two matching water bottles for a day. Subsequently, each mouse underwent a 24-hour period of water and food deprivation, after which they were given access to a 2% sucrose solution in water. After 24 h, the bottles were weighed, and sucrose preference was determined by calculating the ratio of sucrose water intake to the total fluid consumption (sucrose water intake + pure water intake) and then multiplying by 100%.

### Mouse PET experiment

Prior to PET imaging, the mice were fasted for 12 h to ensure the accuracy of the results. Subsequently, they received an injection of 200µCi ^18^F-FDG into the tail vein to track glucose metabolism. After injection, mice were kept under 1.5% isoflurane anesthesia for 1 h to maintain stability. Positioning followed for proper scanning posture. A static scan was performed using the InliView-3000B scanner for 10 min to obtain clear PET images, followed by a CT scan for anatomical details. CT settings were 50 kV, 0.5 mA, and 80ms exposure time. PET images were reconstructed in ListMode with a 140 × 140 × 140 matrix, 40 iterations, and 0.8 mm slice thickness. CT images were reconstructed using the FDK algorithm with a 512 × 512 × 512 matrix, 0.1367 pixel size, and 0.18 mm slice thickness. PMOD software was used to process images, quantify brain region values, and calculate standardized uptake values (SUVs) from the PET images.

### Histopathological analysis

Tissues from mice, comprising brains and noses, were immersed in 4% paraformaldehyde for a period of 48 h. Brains were dehydrated through a graded alcohol series (75%, 80%, 95%, 100%), cleared in dimethylbenzene, paraffin-embedded, and sectioned. Sections were then dewaxed, deparaffinized, and stained with Nissl and hematoxylin-eosin (HE). The stained sections were dehydrated, cleared, and examined using an optical microscope. Nose tissues, after fixation, were decalcified in ethylenediaminetetraacetic acid for one week, paraffin-embedded, and sectioned at 4 μm. These sections were stained with HE and periodic acid-Schiff (PAS). Eosinophil and goblet cell counts were assessed in three randomly selected high-power fields (400×) under a microscope.

### Immunofluorescence assay

Hippocampus tissues were lysed in RIPA buffer with phenylmethylsulfonyl fluoride and protease inhibitors. Post centrifugation at 12,000 rpm at 4 °C for 15 min, supernatants were harvested. Hippocampus tissues were preserved in 4% paraformaldehyde for 48 h, then embedded in paraffin and cut into 5 μm sections. After blocking with 10% goat serum for 30 min, the sections were incubated with primary antibodies (IBA-1, HMGB1, Notch1, Hes-1; all 1:500 dilution from Abcam) overnight at 4 °C. The next day, they were incubated with anti-mouse secondary antibodies (ThermoFisher, 1:800) for 1 h at room temperature, rinsed with PBS, and stained with DAPI. Confocal microscopy was used to capture fluorescent images, and three randomly selected high-power fields (400×) per section were evaluated by two independent, blinded observers. Mean fluorescence intensity of target proteins was quantified according to the guidelines using ImageJ software, version 1.52a (NIH, USA).

### Cell culture and intervention

Magnetic bead sorting preserves the in vivo status of microglia and reflects their relationship with neuroinflammation. As documented in references (Nikodemova and Watters 2012; Liu et al. 2021), the isolation of microglia from the hippocampus was achieved after the establishment of the model. The hippocampal tissue was enzymatically digested, filtered through 70-µm cell strainers to obtain a single-cell suspension, and microglia were sorted using CD11b magnetic beads (Miltenyi Biotec, Germany). After resuspension, loading, and washing, the positive cells were collected from the sorting column. Then the cells were resuspended in medium containing 10% fetal bovine serum (Gibco, USA), plated on polylysine-coated culture plates, and cultured in a humidified 5%CO_2_ incubator at 37 °C.

Firstly, the cells were divided into four groups: Ctrl, CRS, CRS + siRNA-HMGB1, and CRS + siRNA-NC. Microglia were extracted from the hippocampus of control mice for the Ctrl group and CRS mice for the other groups. The Ctrl and CRS groups were untreated, while the CRS + siRNA-HMGB1 and CRS + siRNA-NC groups were transfected with their respective siRNAs under standard culture conditions.

In the second experiment, the groups were assigned to receive CRS, CRS + low-dose metformin (CRS + Met-L), CRS + medium-dose metformin (CRS + Met-M), or CRS + high-dose metformin (CRS + Met-H). Metformin concentrations were 0.02, 0.2, and 2 mmol/L for low, medium, and high doses, respectively, with consistent culture conditions. After the experiment, supernatants and adherent cells were collected from each group for analysis.

In the third experiment, the cell overexpression studies included CRS + Met, CRS + Met + OE-HMGB1 (HMGB1 overexpression), and CRS + Met + OE-NC (empty vector overexpression). Microglia from CRS mice were cultured with 0.2 mmol/L metformin. The CRS + Met group received only metformin, while the CRS + Met + OE-HMGB1 and CRS + Met + OE-NC groups were transfected with the HMGB1 plasmid (pcDNA-HMGB1) and empty vector (pcDNA-NC), respectively, under some culture conditions.

### siRNA and plasmid transfection

Microglia were planted into 6-well plates at a concentration of 5 × 10^5^ per well, ensuring even distribution. Gene silencing procedures were initiated once the cell confluence reached approximately 70–90%, as observed under a microscope. siRNAs (against HMGB1,5’-UCUUGACCACAGAUCUUAATT-3’, Genechem, China) or plasmids were mixed with Lipofectamine 2000 (Invitrogen, USA) in serum-free medium (Gibco, USA). The medium was replaced for the cells in the plate during the resting period. During the resting phase, the culture medium for the plated cells was refreshed. Post a 15-minute interval, 200 µL of the solution was dispensed into each well, and the 6-well plates were marked, gently agitated, and returned to the incubator. Following a 24-hour period, the cell density and condition were assessed. Subsequently, 1 mL of complete growth medium was added to each well for cell nourishment and incubation.

### Western blot

Hippocampus tissues and microglial cells were processed in RIPA buffer, supplemented with protease and phosphatase inhibitors, to isolate total proteins. The protein concentration was determined using the BCA protein ssay kit (Solarbio, China). An equal amount of protein lysates (40 µg) was loaded into 10% SDS polyacrylamide, transferred onto a PVDF membrane, and then blocked in 5% non-fat milk for 1 h. The membranes were incubated overnight at 4 ℃ with the following primary antibodies: HMGB1 (1:1000, Abcam), Notch1 (1:1000, Abcam), Hes-1 (1:1000, Abcam) and GAPDH (1:1000, Abcam). The membrane was incubated with secondary goat anti-rabbit antibodies (1:3000, Abcam) for 1 h at room temperature after washing with TBS and 0.05% Tween-20. Protein levels were normalized to GAPDH and quantified using ImageJ software, version 1.52a (NIH, USA).

### Quantitative real-time PCR analysis

Total cellular RNA from the samples was isolated using RNA isolation reagent (Servicebio, China), following the manufacturer’s instructions. The concentration and purity of the RNA were assessed using a spectrophotometer. A quantity of 1 µg of RNA was then reverse-transcribed into cDNA using the PrimeScript™ RT reagent Kit with gDNA Eraser (Takara, China), which served as a template for PCR. Quantitative real-time PCR was performed using the TB Green^®^ Premix Ex Taq™ II (Takara, China) with specific primers according to the manufacturer’s protocol. The primer sequences are as follows:

HMGB1: forward, 5’- CACCGTGGGACTATTAGGAT − 3’; reverse, 5’- GCTCACACTTTTGGGGATAC − 3’; Notch1: forward, 5’- TGCCAGTATGATGTGGATGAG − 3’; reverse, 5’- GGTCCCTGTGTAACCTTCTGT − 3’; Hes-1: forward, 5’- AGCCCACCTCTCTCTTCTGAC − 3’; reverse, 5’- AGGCGCAATCCAATATGAAC − 3’; GAPDH: forward, 5’- GCCTCGTCTCATAGACAAGATG − 3’; reverse, 5’- CAGTAGACTCCACGACATAC − 3’. GAPDH served as an endogenous control. Relative levels of mRNA were presented as the relative fold change and calculated using the 2^−△△CT^ method.

### Enzyme linked immunosorbent assay (ELISA)

After drug treatments, hippocampus tissues and culture media were collected from the mice. Tissues from both hippocampi were homogenized, and 200 µL of the lysate was added. The solution was chilled on ice for 30 min prior to centrifugation at 12,000 rpm for a duration of 5 min. The supernatants from both the lysate and the culture media were retained for analysis. ELISA kits specific for mice (Servicebio, China) were used to determine the levels of TNF-α, interleukin (IL)-1β, and IL-6, following the manufacturer’s instructions.

### Statistical analysis

All experimental data are expressed as mean ± standard deviation. To determine statistical differences between pairs of groups, Student’s t-test was applied. For comparisons involving more than two groups, one-way analysis of variance was conducted, with subsequent analysis by Bonferroni’s post hoc test. Statistical significance was set at a p-value of less than 0.05. The software GraphPad Prism 8.0 (GraphPad Software, USA) was utilized for all statistical analyses.

## Results

### The impact of CRS with varying degrees of inflammation on anxiety and depression symptoms in CRS mice

**Fig. 2:**
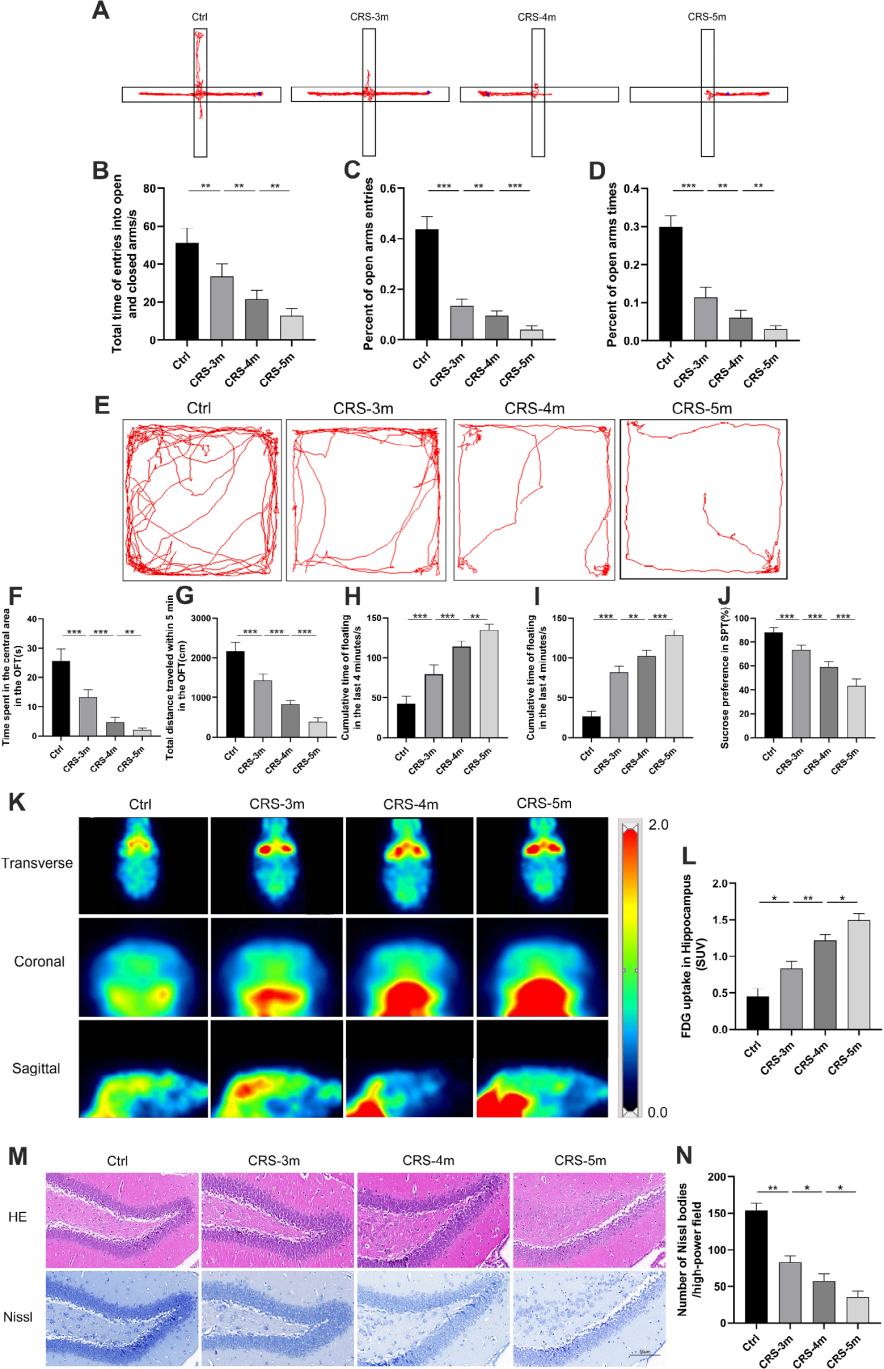
Anxiety and depression-like behaviors in CRS with varying inflammation. (**A**) Elevated plus maze test track plot. (**B**) Open and closed arm time for mice. (**C**-**D**) Open arm entries and time spent proportions. (**E**) Open field test track plot. (**F**) Time in central zone. (**G**) Total distance traveled. (**H**) Tail suspension test immobility time. (**I**) Forced swim test immobility time. (**J**) Sucrose preference test value. (**K**) Mouse brain PET images. (**L**) Hippocampal region SUV statistics. (**M**) Hippocampal region histopathological staining (×200). (**N**) Hippocampal Nissl body count. Data are presented as mean ± SD. *P < 0.05. **P < 0.01, ***P < 0.001

CRS mouse models with different durations were established (Fig. 1A). The models’ validity was confirmed by assessing eosinophil infiltration and goblet cell hyperplasia in the nasal mucosa. Compared to the Ctrl group, the CRS group showed a significant increase in goblet cells and eosinophil infiltration (Fig. 1B-D). The anxiety and depressive symptoms of Mice were evaluated using various behavioral tests (Fig. 1E). Furthermore, the CRS-5 m group showed a notably higher number of eosinophils and goblet cells in the nasal mucosa compared to the CRS-3 m and CRS-4 m groups (Fig. 1F-G). In the elevated plus maze, the CRS-3 m group spent significantly less time in the open arms and made fewer entries compared to the Ctrl group (Fig. 2A-D). The CRS-5 m group showed an even greater decrease in these parameters compared to both the CRS-3 m and CRS-4 m groups. In the open field test, the CRS group spent less time in the central area and covered less distance than the Ctrl group, with the CRS-5 m group showing the most significant reduction compared to the CRS-3 m and CRS-4 m groups (Fig. 2E-G). The tail suspension test revealed that the CRS-5 m group had the longest cumulative immobility time in the last 4 min (Fig. 2H). Similarly, in the forced swim test, the CRS-5 m group had a significantly longer cumulative immobility time than the Ctrl, CRS-3 m, and CRS-4 m groups in the last 4 min (Fig. 2I). Lastly, the sucrose preference test indicated that the CRS-5 m group had a significantly lower preference rate for sucrose compared to the other groups (Fig. 2J).

### The impact of CRS with different inflammatory degrees on the hippocampal region of CRS mice

To evaluate the impact of CRS on hippocampal metabolism, ^18^F-FDG PET scans and SUV calculations were performed. The findings revealed that the hippocampal SUV was markedly elevated in the CRS-3 m group compared to the Ctrl group, with the CRS-5 m group showing an even higher SUV than both the CRS-3 m and CRS-4 m groups (Figures K-L). This indicates that increased CRS inflammation correlates with higher metabolic activity in the hippocampus.

**Fig. 3:**
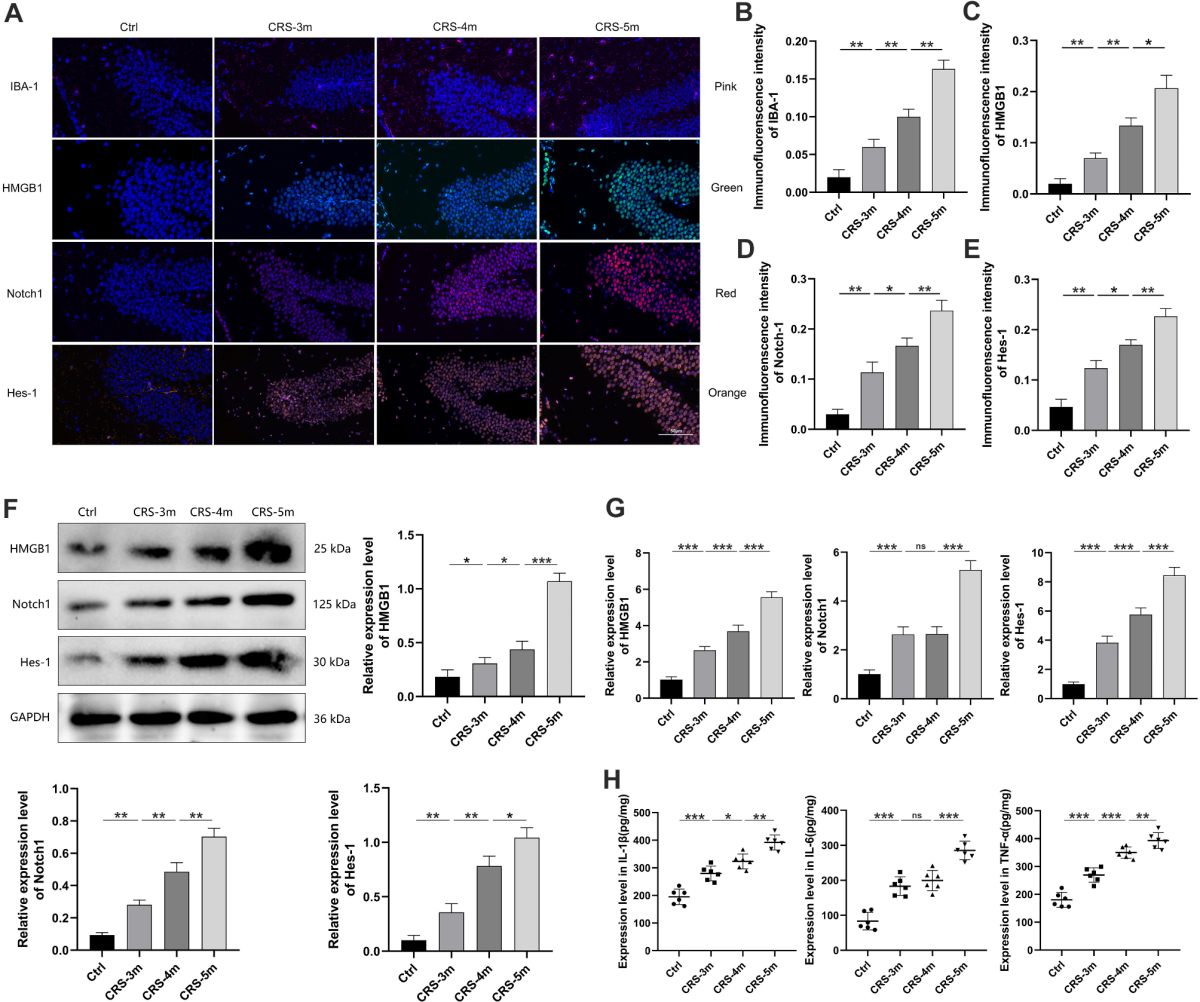
CRS Effects on hippocampal HMGB1, Notch1, and Hes-1 expression in mice. (**A**) Immunofluorescence of the hippocampus: DAPI (blue), IBA-1 (pink), HMGB1 (green), Notch1 (red), Hes-1 (orange); merged images (×200). (**B**-**E**) Immunofluorescence intensities for IBA-1, HMGB1, Notch1, and Hes-1. (**F**) Normalized hippocampal protein expression of HMGB1, Notch1, and Hes-1. (**G**) Hippocampal mRNA expression of HMGB1, Notch1, and Hes-1. (**H**) Hippocampal expression of IL-1β, IL-6, and TNF-α. Data are presented as mean ± SD. ns: no signifcance; *P < 0.05. **P < 0.01, ***P < 0.001

HE staining showed no significant morphological changes in the hippocampal structure across all mouse groups (Fig. 2M). Nissl staining revealed a significant reduction in the number of Nissl bodies and lighter staining in hippocampal neurons of the CRS-3 m group compared to the Ctrl group, with the CRS-5 m group exhibiting an even more pronounced decrease and lighter staining compared to the CRS-3 m and CRS-4 m groups (Fig. 2N). Immunofluorescence analysis demonstrated significant upregulation of IBA-1, HMGB1, Notch1, and Hes-1 in the hippocampal region of the CRS-3 m group relative to the Ctrl group, with the CRS-5 m group showing higher expression levels than both the CRS-3 m and CRS-4 m groups (Fig. 3A-E).

Additionally, Western Blot and RT-qPCR were used to quantify the level of HMGB1, Notch1, and Hes-1, while ELISA measured inflammatory factors. The findings indicated that the hippocampal levels of HMGB1, Notch1, Hes-1, IL-1β, IL-6, and TNF-α in the CRS-3 m group markedly exceeded those in the Ctrl group. Furthermore, the CRS-5 m group showed upregulated expression of these molecules compared to both the CRS-3 m and CRS-4 m groups (Fig. 3F-H).

### Inhibiting the expression of HMGB1 alleviates symptoms of anxiety and depression in CRS mice

**Fig. 4:**
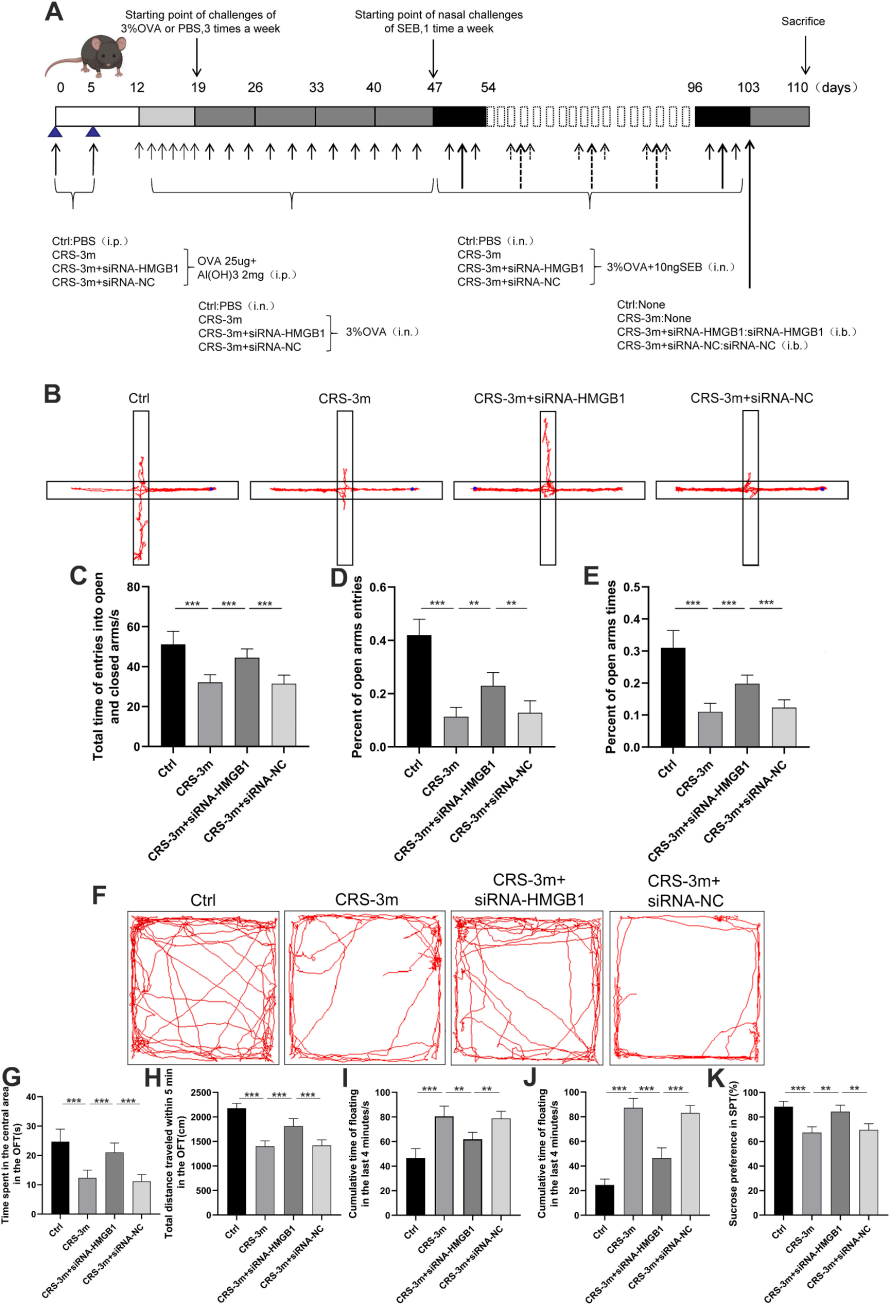
Inhibition of HMGB1 reduces anxiety and depression in CRS mice. (**A**) Experimental procedure timeline. (**B**) Elevated plus maze test track plot. (**C**) Time in open and closed arms. (**D**-**E**) Open arm entries and time spent proportions. (**F**) Open field test track plot. (**G**) Time in central zone. (**H**) Total distance traveled. (**I**) Tail suspension test immobility time. (**J**) Forced swim test immobility time. (**K**) Sucrose preference test value. Data are presented as mean ± SD. **P < 0.01, ***P < 0.001

Stereotactic injection of HMGB1-siRNA into the hippocampus was performed to assess its effects on CRS mice (Fig. 4A). In the elevated plus maze, CRS-3 m group showed reduced time and entries in the open arms and a lower proportion of time spent there compared to the Ctrl group; these behaviors were significantly improved in the CRS-3 m + siRNA-HMGB1 group (Fig. 4B-E). In the open field test, CRS-3 m group allocated a shorter duration to the central zone and traveled a shorter distance, which were partially restored in the CRS-3 m + siRNA-HMGB1 group (Fig. 4F-H). The tail suspension test showed increased immobility in the CRS-3 m group, which was reduced in the CRS-3 m + siRNA-HMGB1 group (Fig. 4I). Similarly, the forced swim test indicated a decrease in immobility time in the CRS-3 m + siRNA-HMGB1 group (Fig. 4J). Lastly, the sucrose preference test revealed a higher sucrose preference in the CRS-3 m + siRNA-HMGB1 group compared to the CRS-3 m group (Fig. 4K).

### Inhibiting HMGB1 expression in CRS mice affected hippocampal metabolism and neuronal damage

**Fig. 5:**
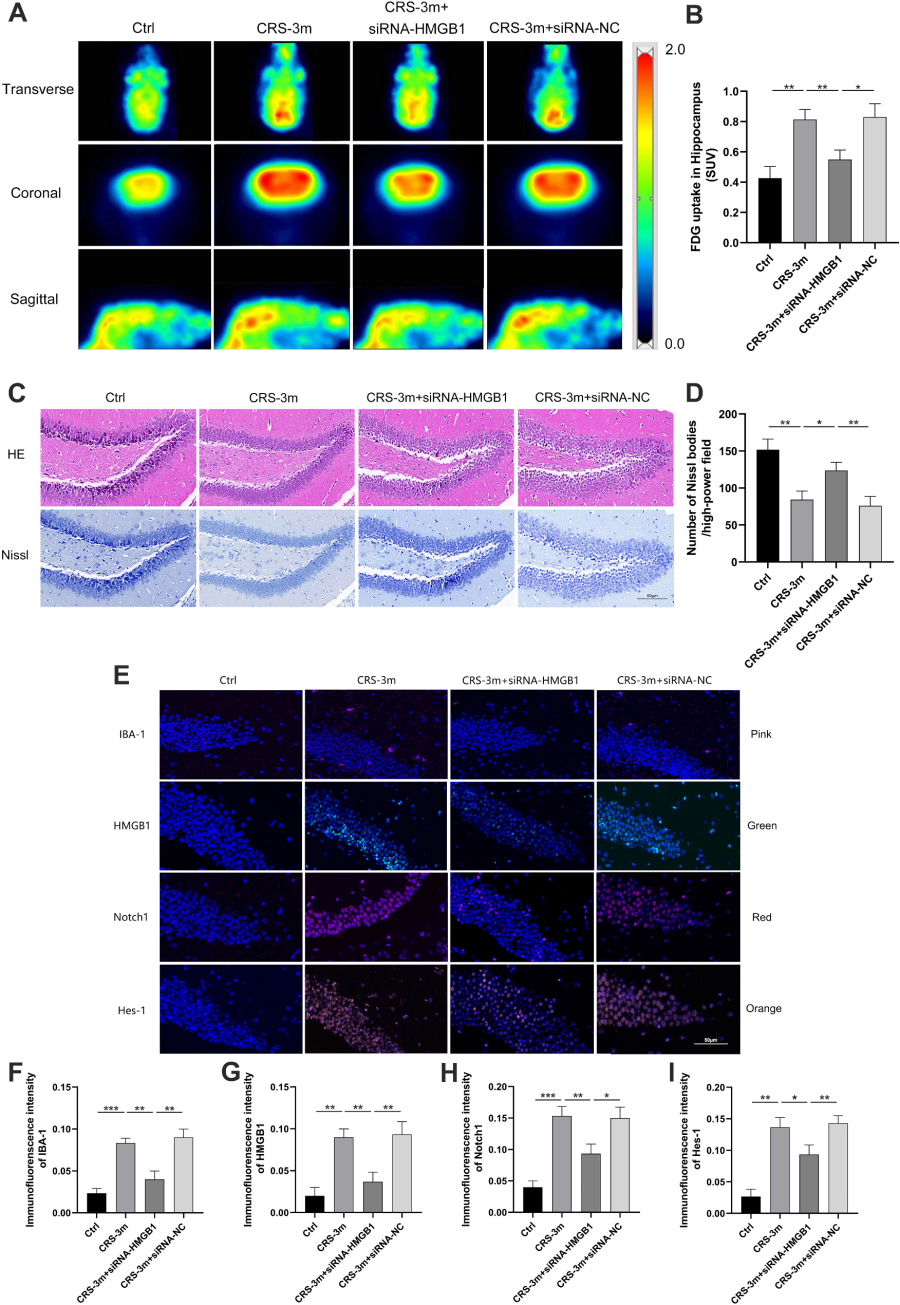
Impact of HMGB1 inhibition on metabolism and neuronal damage in the hippocampus of CRS mice. (**A**) Mouse brain PET images. (**B**) Hippocampal SUV statistics. (**C**) Hippocampal histopathological staining (×200). (**D**) Hippocampal Nissl body count. (**E**) Hippocampal immunofluorescence images (×200). (**F**-**I**) Immunofluorescence intensities for IBA-1, HMGB1, Notch1, and Hes-1. Data are presented as mean ± SD. *P < 0.05. **P < 0.01, ***P < 0.001

**Fig. 6:**
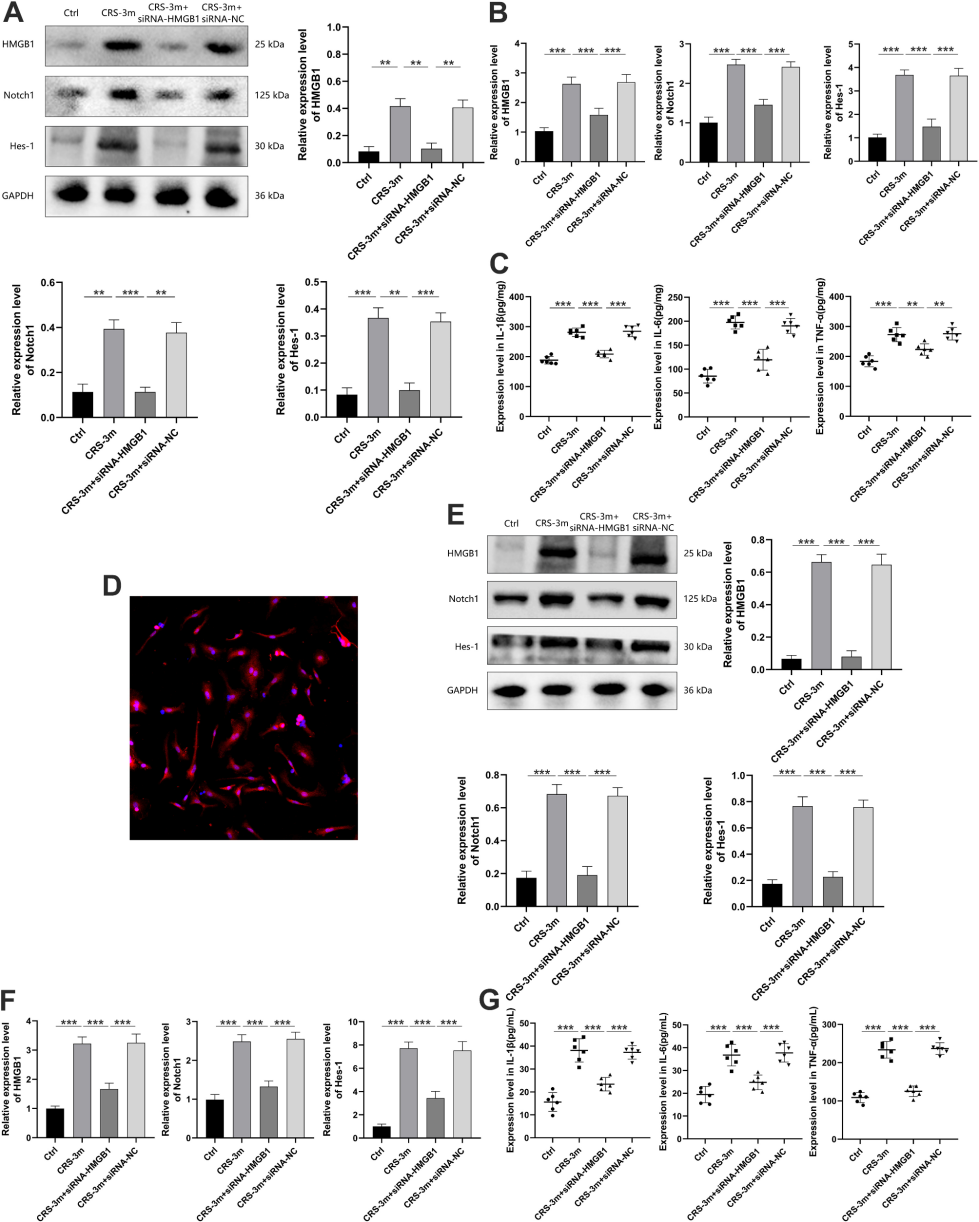
Impact of HMGB1 inhibition on the Notch1/Hes-1 Pathway. (**A**) Hippocampal protein expression of HMGB1, Notch1, and Hes-1. (**B**) Hippocampal mRNA expression of HMGB1, Notch1, and Hes-1. (**C**) Hippocampal expression of IL-1β, IL-6, and TNF-α. (**D**) Primary microglia labeled by CD11b (×200). (**E**) Microglial protein expression of HMGB1, Notch1, and Hes-1. (**F**) Microglial mRNA expression of HMGB1, Notch1, and Hes-1. (**G**) Expression levels of IL-1β, IL-6, and TNF-α. Data are presented as mean ± SD. **P < 0.01, ***P < 0.001

Compared to the CRS-3 m group, the hippocampal SUV in the CRS-3 m + siRNA-HMGB1 group was significantly reduced (Fig. 5A-B). HE staining indicated no structural differences between groups, while Nissl staining revealed a significant reduction in Nissl bodies and lighter staining in the CRS-3 m group, which were partially restored in the CRS-3 m + siRNA-HMGB1 group (Fig. 5C-D). Immunofluorescence showed upregulated IBA-1, HMGB1, Notch1, and Hes-1 in the CRS-3 m group, with downregulation in the CRS-3 m + siRNA-HMGB1 group (Fig. 5E-I). Furthermore, the hippocampal expression of HMGB1, Notch1, along with key inflammatory cytokines, were upregulated in the CRS-3 m group and downregulated in the CRS-3 m + siRNA-HMGB1 group (Fig. 6A-C).

### The impact of inhibiting HMGB1 expression on the Notch1/Hes-1 pathway in mouse microglia

Primary microglia were extracted for in vitro experiments to explore the molecular mechanisms (Fig. 6D). The results indicated that the CRS-3 m group had significantly higher expression of HMGB1, Notch1, Hes-1, IL-1β, IL-6, and TNF-α than the Ctrl group. The CRS-3 m + siRNA-HMGB1 group showed reduced levels of these markers compared to the CRS-3 m group (Fig. 6E-G).

### Metformin mitigates symptoms of anxiety and depression in CRS mice

**Fig. 7:**
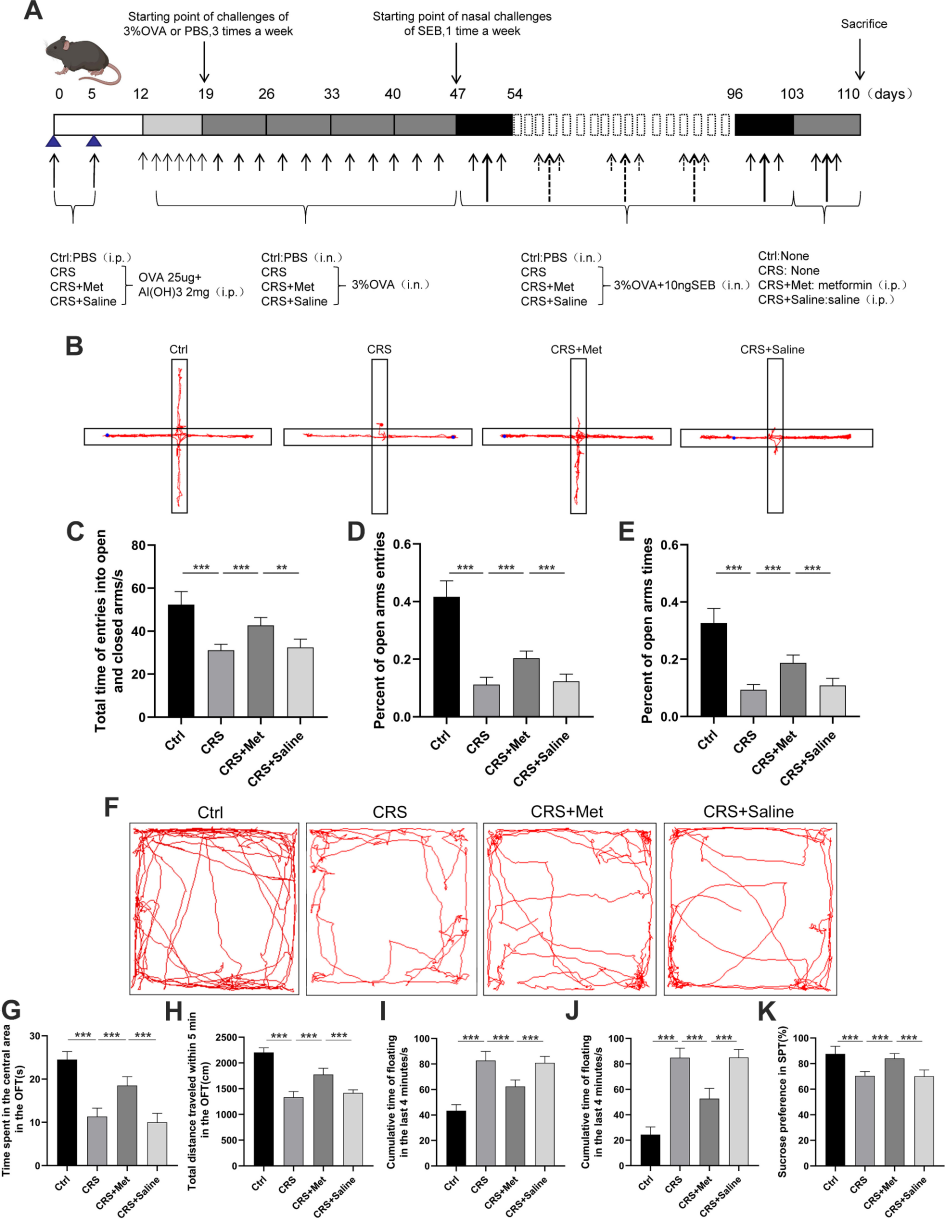
Effect of metformin on anxiety and depression in CRS mice. (**A**) Experimental procedure timeline. (**B**) Elevated plus maze test track plot. (**C**) Time in open and closed arms. (**D**-**E**) Open arm entries and time spent proportions. (**F**) Open field test track plot. (**G**) Time in central zone. (**H**) Total distance traveled. (**I**) Tail suspension test immobility time. (**J**) Forced swim test immobility time. (**K**) Sucrose preference test value. Data are presented as mean ± SD. **P < 0.01, ***P < 0.001

Metformin was administered intraperitoneally to CRS mice to assess its effects on symptoms of anxiety and depression (Fig. 7A). Compared to the Ctrl group, CRS mice exhibited reduced time, frequency, and ratio of time in the open and closed arms; these behaviors improved significantly after metformin treatment (CRS + Met group) (Fig. 7B-E). CRS mice demonstrated reduced time spent in the central area and covered a shorter distance compared to the Ctrl group; metformin treatment increased these indicators significantly (Fig. 7F-H). The tail suspension test showed longer periods of immobility in CRS mice, a duration that was reduced following metformin administration (Fig. 7I). Similarly, the forced swim test indicated that the CRS + Met group exhibited less immobility time compared to the CRS group (Fig. 7J). Lastly, the sucrose preference test revealed a marked increase in sucrose preference for the CRS + Met group compared to the CRS group (Fig. 7K).

### Metformin reduces metabolic levels and neuronal damage in the hippocampus of CRS mice

**Fig. 8:**
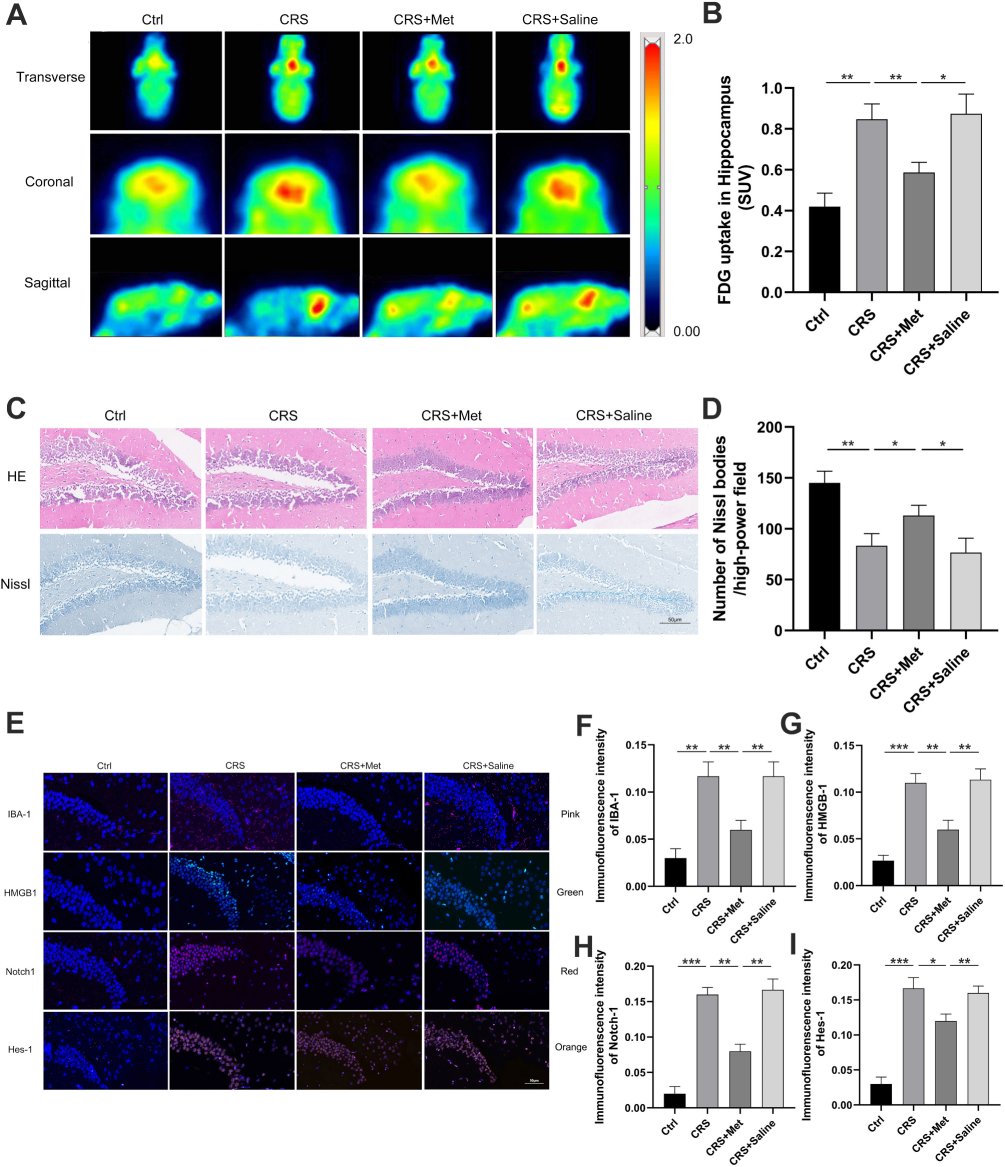
Metformin decreases metabolic levels and neuronal injury in CRS mouse hippocampus. (**A**) Mouse brain PET images. (**B**) Hippocampal SUV statistics. (**C**) HE-stained and Nissl-stained hippocampal images (×200). (**D**) Hippocampal Nissl body count. (**E**) Hippocampal immunofluorescence images (×200). (**F**-**H**) Immunofluorescence intensities for IBA-1, HMGB1, Notch1, and Hes-1. Data are presented as mean ± SD. *P < 0.05. **P < 0.01, ***P < 0.001

The hippocampal SUV was higher in the CRS group than in the Ctrl group and lower in the CRS + Met group than in the CRS group (Fig. 8A-B). While HE staining did not reveal any marked differences in hippocampal structure across the groups, Nissl staining revealed lighter and fewer Nissl bodies in the CRS group, whereas the CRS + Met group showed a denser and more abundant staining (Fig. 8C-D).

### The impact of inhibiting HMGB1 expression on metabolic levels and neuronal damage in the hippocampus of CRS mice

**Fig. 9:**
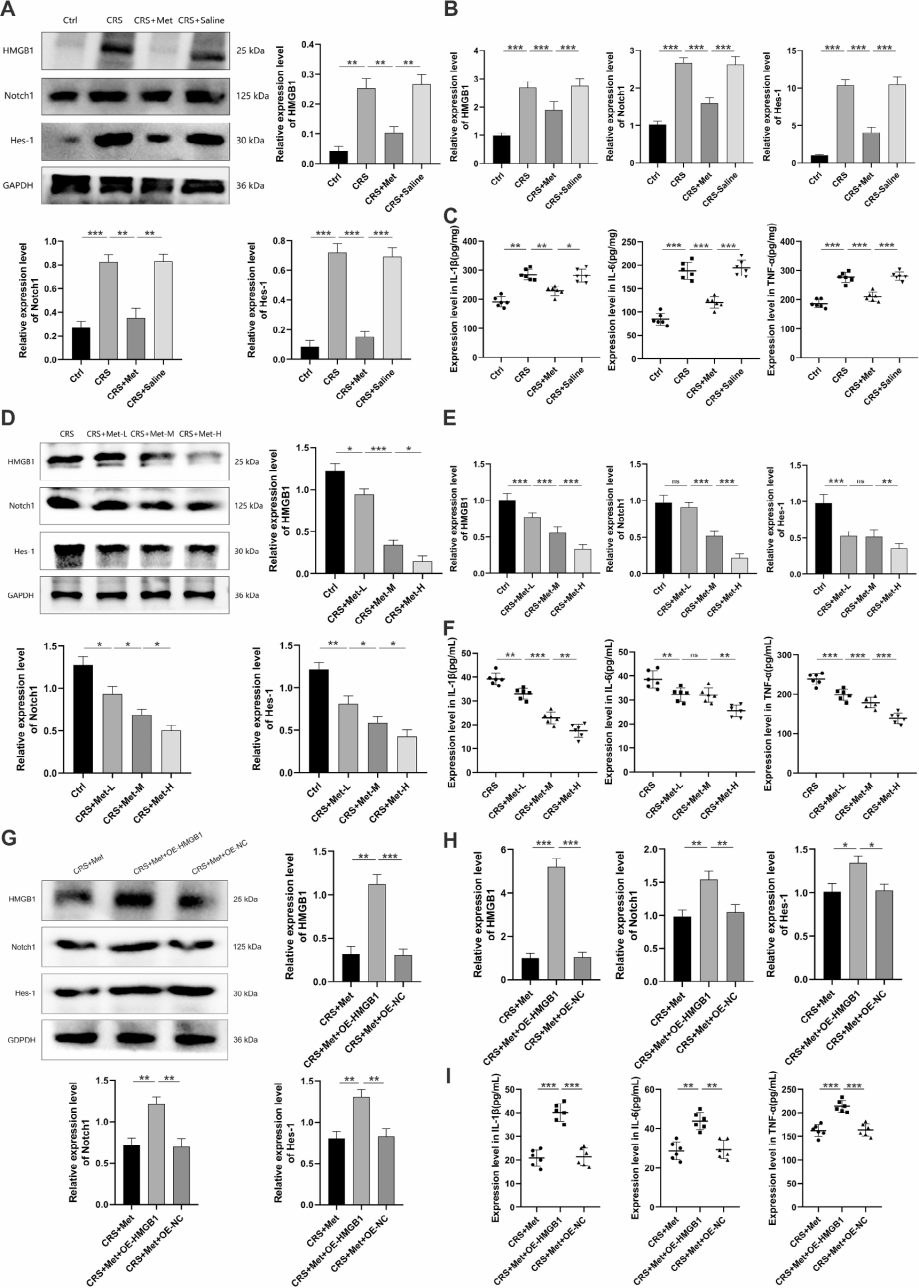
Metformin inhibits HMGB1-mediated activation of the Notch1/Hes-1 pathway in CRS mouse hippocampus. (**A**) Hippocampal protein expression of HMGB1, Notch1, and Hes-1. (**B**) Hippocampal mRNA expression of HMGB1, Notch1, and Hes-1. (**C**) Hippocampal expression of IL-1β, IL-6, and TNF-α. (**D**) Microglial protein expression of HMGB1, Notch1, and Hes-1 post-metformin. (**E**) Microglial mRNA expression of HMGB1, Notch1, and Hes-1. (**F**) Expression levels of IL-1β, IL-6, and TNF-α. (**G**) Microglial protein expression of HMGB1, Notch1, and Hes-1 post-siRNA. (**H**) Microglial mRNA expression of HMGB1, Notch1, and Hes-1 post-siRNA. (**I**) Expression levels of IL-1β, IL-6, and TNF-α. Data are presented as mean ± SD. ns: no signifcance; *P < 0.05; **P < 0.01; ***P < 0.001

Immunohistochemical analysis revealed increased IBA-1, HMGB1, Notch1, and Hes-1 expression in the hippocampus of the CRS mice versus the Ctrl group, whereas the CRS + Met group showed reduced expression (Fig. 8E-I). Hippocampal levels of HMGB1, Notch1, Hes-1, TNF-α, IL-1β, and IL-6 were elevated in the CRS group and lower in the CRS + Met group (Fig. 9A-C). In vitro cellular experiments indicate that increasing metformin concentrations can reduce the expression of these factors (Fig. 9D-F). Overexpressing HMGB1 heightened their expression levels (Fig. 9G-I), suggesting that HMGB1 overexpression counteracts metformin’s anti-inflammatory effects.

## Discussion

Considerable evidence links CRS with anxiety and depressive disorders, but the mechanisms are not fully understood. Firstly, patients with CRS often face psychological impacts like sleep disturbances, pain, and cognitive difficulties, potentially raising their susceptibility to depression or anxiety (Alt et al. 2013). Secondly, sinonasal symptoms like impaired smell or taste and nasal congestion may lead to social isolation, further elevating likelihood of depression and anxiety. Like other chronic conditions, CRS requires substantial time for medical consultations and treatments like nasal irrigation, potentially leading to frustration and the onset of depression or anxiety (Schlosser et al. 2016). Additionally, CRS patients may develop depression as a side effect of medications, with frequent glucocorticoid use associated with increased depressive or manic symptoms and behavioral changes (Brown and Chandler 2001). An hypothesis of systemic inflammation has been put forward for various chronic conditions, suggesting a correlation between inflammatory cytokine levels and depression severity, as seen in multiple sclerosis, asthma, rheumatic diseases, and allergic rhinitis (Felger and Lotrich 2013). Nevertheless, this hypothesis remains uninvestigated in the context of CRS. Additional research is required to elucidate the link between CRS and disorders characterized by anxiety and depression.

This study reveals that CRS mice exhibit increased anxiety and depression-like behaviors correlated with CRS severity. High HMGB1 expression in the hippocampi of these mice was observed, and alleviation of these behaviors followed the stereotactic injection of HMGB1-siRNA into their hippocampal regions. Cellular experiments indicated that the Notch1/Hes-1 signaling pathway and microglial inflammatory factors were significantly upregulated in the hippocampi of CRS mice, with siRNA intervention reducing these expressions. HMGB1 appears to trigger neuroinflammation by activating the Notch1/Hes-1 pathway in hippocampal microglia, contributing to the emergence of anxiety and depression-like symptoms. The hippocampus, part of the limbic system and crucial for social memory formation and storage, is also integral to the development of emotional disorders like anxiety and depression (Zhong et al. 2019). It regulates learning, memory, and cognition, with structural or functional alterations potentially leading to depressive and anxiety-related symptoms (Kumaran and Maguire 2005). Normally, the brain maintains a self-regulating homeostasis, but quiescent microglia activate in response to external stimuli or injury, clearing apoptotic nerve cells and pathogenic factors, which is vital for immune defense (Liu 2006). This study confirms that CRS-related anxiety-depressive symptoms are closely linked to neuroinflammation mediated by activated hippocampal microglia.

The Notch signaling pathway is essential for CNS physiological functions, brain development, synaptic plasticity, neuron survival, and the regulation of neural stem cell proliferation and differentiation (Jin et al. 2019). It also contributes to neuroinflammation in various diseases (Zhang et al. 2022). In knockout mice, activated microglia and inflammatory factor levels in the ischemic cortex are significantly lower than in wild-type mice (Chen et al. 2020). This study found that CRS mice with severe anxiety-depressive symptoms had significantly increased hippocampal expression levels of Notch1/Hes-1, indicating the importance of microglial Notch1/Hes-1 pathway activation. Longer CRS modeling times correlated with higher hippocampal HMGB1 and Notch1/Hes-1 pathway protein expressions, suggesting a link between CRS severity and neuroinflammation. siRNA knockdown of HMGB1 in microglia significantly reduced Notch1/Hes-1 pathway proteins and inflammatory factors, highlighting HMGB1’s role in activating the Notch1/Hes-1 pathway and contributing to anxiety-depressive-like behaviors in CRS mice. In neuroinflammation, IL-1β and IL-6 are key cytokines that promote neuronal vitality and function at low concentrations but damage synaptic plasticity at high concentrations, contributing to the pathological processes of neurodegenerative and inflammatory diseases (Kempuraj et al. 2016). TNF-α, a potent immuno-activating factor, is released in excessive amounts by microglia cells under pathological conditions, inducing inflammatory cascade reactions in the CNS (Liu et al. 2017). In this study, compared to the control group, CRS mice with anxiety and depression showed significantly increased expression levels of Notch1/Hes-1, TNF-α, IL-1β, and IL-6 in the hippocampal region. Neuroinflammation is a complex network involving the interplay of various cells and molecules. In the hippocampal region, neuroinflammation may not be solely caused by the activation of a single type of cell, but rather may be an effect driven by the interaction between neurons and microglia. In subsequent research, we still need to explore the role of HMGB1 in neurons and its interaction with microglia.

This study confirms that CRS mice exhibit symptoms of anxiety and depression, with elevated HMGB1 expression and stimulation of the Notch1/Hes-1 pathway in hippocampal microglia. Metformin is a first-line drug for the treatment of type 2 diabetes, and it can penetrate the blood-brain barrier to act directly on the brain, playing an important role in the treatment of neurodegenerative diseases and neuroinflammation (Cao et al. 2022). Metformin, when administered intraperitoneally, significantly improved behavioral indices and reduced HMGB1, Notch1/Hes-1 pathway, and inflammatory factor expressions in the hippocampus. In vitro experiments further demonstrated that metformin inhibits HMGB1-mediated Notch1/Hes-1 pathway activation in microglia and decreases inflammatory factor release. These findings indicate that metformin acts on hippocampal microglia, alleviates CNS inflammation by inhibiting these pathways, and effectively improves anxiety-depressive-like behaviors in CRS mice. Metformin is known for its anti-inflammatory effects in CNS diseases, reducing LPS-induced inflammatory factor expression in PD rats and inhibiting Toll-like receptor-activated microglia (Gil-Martínez et al. 2018). It also alleviates neurofunctional degeneration in AD by regulating multiple hippocampal signaling pathways (Ou et al. 2018) and improves cognitive and motor functions in haloperidol-induced PD mouse models (Yimer et al. 2019). Additionally, it inhibits microglial activation and reduces neuroinflammation in a cerebral hemorrhage mouse model (Yu et al. 2022). This study also verifies that metformin inhibits microglial activation and inflammatory factor release in the hippocampus of CRS mice, thereby improving neuroinflammation.

This study has some limitations. First, in vitro cell experiments cannot fully simulate the real internal environment of the CNS in humans or mice under CRS conditions, as the network of cytokine interactions involved is extremely complex. Second, a time gradient for metformin treatment of cells was not set up in the cell experiments. Additionally, the drug toxicity of metformin was not assessed in this study. It has been confirmed that metformin pretreatment can reduce inflammation and DNA damage in vitro BV2 cell line (Xiang et al. 2023). The last, this study employed standard behavioral tests to evaluate anxiety and depression in CRS mice. While a Hospital Anxiety and Depression Scale (HADS) score of 11 or higher indicates anxiety or depression in patients, no such threshold exists for mice. Thus, we can only compare symptom scores across groups, not calculate exact incidence rates for these conditions. In future research, we will enhance the experimental design and explore related mechanisms to identify new therapeutic approaches for anxiety and depression symptoms associated with CRS.

## Data Availability

No datasets were generated or analysed during the current study.
